# Estimation of linkage disequilibrium in four US pig breeds

**DOI:** 10.1186/1471-2164-13-24

**Published:** 2012-01-17

**Authors:** Yvonne M Badke, Ronald O Bates, Catherine W Ernst, Clint Schwab, Juan P Steibel

**Affiliations:** 1 Department of Animal Science, Michigan State University, East Lansing, MI, USA; 2 Department of Fisheries and Wildlife, Michigan State University, East Lansing, MI, USA; 3 National Swine Registry, West Lafayette, IN, USA

## Abstract

**Background:**

The success of marker assisted selection depends on the amount of linkage disequilibrium (LD) across the genome. To implement marker assisted selection in the swine breeding industry, information about extent and degree of LD is essential. The objective of this study is to estimate LD in four US breeds of pigs (Duroc, Hampshire, Landrace, and Yorkshire) and subsequently calculate persistence of phase among them using a 60 k SNP panel. In addition, we report LD when using only a fraction of the available markers, to estimate persistence of LD over distance.

**Results:**

Average *r^2 ^*between adjacent SNP across all chromosomes was 0.36 for Landrace, 0.39 for Yorkshire, 0.44 for Hampshire and 0.46 for Duroc. For markers 1 Mb apart, *r^2 ^*ranged from 0.15 for Landrace to 0.20 for Hampshire. Reducing the marker panel to 10% of its original density, average *r^2 ^*ranged between 0.20 for Landrace to 0.25 for Duroc. We also estimated persistence of phase as a measure of prediction reliability of markers in one breed by those in another and found that markers less than 10 kb apart could be predicted with a maximal accuracy of 0.92 for Landrace with Yorkshire.

**Conclusions:**

Our estimates of LD, although in good agreement with previous reports, are more comprehensive and based on a larger panel of markers. Our estimates also confirmed earlier findings reporting higher LD in pigs than in American Holstein cattle, especially at increasing marker distances (> 1 Mb). High average LD (*r^2 ^*> 0.4) between adjacent SNP found in this study is an important precursor for the implementation of marker assisted selection within a livestock species.

Results of this study are relevant to the US purebred pig industry and critical for the design of programs of whole genome marker assisted evaluation and selection. In addition, results indicate that a more cost efficient implementation of marker assisted selection using low density panels with genotype imputation, would be feasible for these breeds.

## Background

The extent of non-random association of gametes at different loci, or linkage disequilibrium (LD), has become the focus of many recent studies in both humans and animals [1-4]. Gaining knowledge of the distribution of LD in livestock populations is important for genetic mapping of economically important traits such as disease resistance [5], and it can reveal population history and breed development [6,7]. Moreover, genome wide association (GWAs) studies as well as genomic selection in livestock rely on the existence of LD between causative variants and genetic markers [8,9]. Recent advances in genotyping technology allow high density genotyping of single nucleotide polymorphisms (SNP) for several livestock species such as cattle [10], chickens [11] and pigs [12]. Obtaining high density genotypes from a sample of individuals allows for the estimation of genome-wide LD and persistence of phase among breeds [13].

Previous studies have shown that the extent and persistence of LD in livestock [14-16] is much larger than that found in human populations [3], due to selection and smaller effective population size in livestock species [1,17]. Using dense markers to cover the genome increases the likelihood of SNP markers to be in high LD with causative genes altering important production phenotypes [18]. Meuwissen et al. [19] proposed that the merit of these markers in livestock would be in the parallel use of all markers to derive genomic breeding values (GEBV) as a composite score of all individual SNP effects rather than improving mapping of quantitative trait loci (QTL).

The implementation of genomic selection using GEBV has been successful in dairy cattle [8,20,21], and is currently being tested in laying chickens [22], and pigs [23]. The reliability of GEBV prediction relies on the level of LD between markers and QTL, the origin of such LD (either within family or population-wise), the number of animals used in the training population as well as heritability of the trait [8]. In this study it is our objective to estimate and describe genome wide levels of LD in four pig breeds using high density genotypes. We also estimate population-wise LD for a variety of panels with lower marker density in order to estimate the number of markers needed to reach a given level of LD. We estimate persistence of phase between the four breeds in this study as a measure of relationship between these populations.

## Results

### Estimation of Linkage Disequilibrium

**Figure 1:**
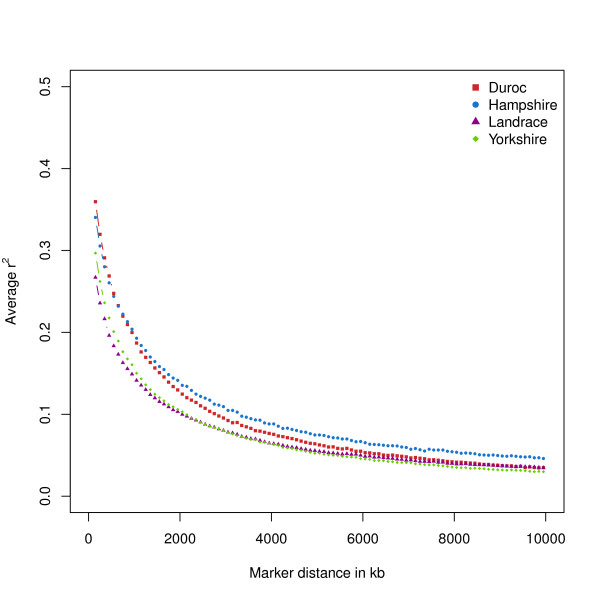
**Decay of average *r^2 ^*over distance**. Average *r^2 ^*between markers in Duroc, Hampshire, Landrace and Yorkshire at various distances in base pairs ranging from 0 to 10 Mb.

To estimate LD, we genotyped 351 animals in 117 sire/dam/offspring trios across four breeds of pigs (Duroc, Hampshire, Landrace and Yorkshire) using the Illumina PorcineSNP60 BeadChip [12]. We used BEAGLE [24] to build haplotypes and estimated pairwise *r^2 ^*for all SNP on the same chromosome using equation (1). Average *r^2 ^*between adjacent markers within breed was estimated using equation (2). Average *r^2 ^*at various distances was computed by grouping all SNP combinations by their pairwise distance in classes of 100 kb of length starting at 0 to 10 Mb. Figure 1 displays an overview of the decline of *r^2 ^*over distance in each breed. In addition, Table 1 displays average *r^2 ^*for adjacent markers and at 0.5, 1 and 5 Mb. The average *r^2 ^*between adjacent SNP was largest in the Duroc animals (*r^2 ^*= 0.46), followed by Hampshire (*r^2 ^*= 0.44), whereas Yorkshire and Landrace exhibited the smallest average *r^2 ^*(0.39 and 0.36 respectively; Table 1). Marker pairs with an average distance of 1 Mb had an average *r^2 ^*of 0.20 for Hampshire, 0.19 for Duroc, 0.16 for Yorkshire and 0.15 for Landrace. For all breeds, at least 54% of the adjacent SNP had *r^2 ^*≥ 0.2 and 44% had *r^2 ^*≥ 0.3. For most chromosomes, average *r^2 ^*between adjacent SNP in Duroc and Hampshire was larger than average *r^2 ^*in Landrace or Yorkshire. In addition to estimating average *r^2 ^*within distance classes, we also computed average *r^2 ^*between adjacent markers for different marker densities. To obtain marker sets with various SNP densities we sequentially removed markers from the current map using every second, fourth, 10^th^, 50^th^, 100^th ^and 200^th ^marker (Table 2). Average *r^2 ^*decreased between 6% for Yorkshire to 15% for Hampshire when only 50% of the markers were used, with highest average *r^2 ^*for Duroc (*r^2 ^*= 0.40) followed by Hampshire (*r^2 ^*= 0.37), Yorkshire (*r^2 ^*= 0.34) and the lowest for Landrace (*r^2 ^*= 0.30). Using only every 10^th ^marker, average *r^2 ^*decreased to around 50% of the original *r^2 ^*(*r^2 ^*= 0.20-0.25), and using every 100^th ^marker average *r^2 ^*ranged from 0.05-0.07 at an average marker distance of 6.5 Mb, which was comparable to the results found for average *r^2 ^*at 5 Mb.

**Table 1:** Average *r^2 ^*at various distances in four breeds

Breed	Adj.^1^	0.5 Mb^2^	1 Mb^2^	5 Mb^2^
Duroc	0.46	0.26	0.19	0.06
Hampshire	0.44	0.25	0.20	0.08
Landrace	0.36	0.19	0.15	0.06
Yorkshire	0.39	0.21	0.16	0.05

^1^Average *r^2 ^*for SNP with adjacent map positions (exact spacing: 70 kb for Duroc, 74 kb for Hampshire, 60 kb for Landrace, and 61 kb Yorkshire).

^2^Average *r^2 ^*for SNP spaced 0.5 Mb, 1 Mb and 5 Mb apart

**Table 2:** Average *r^2 ^*between adjacent SNP for sparse marker panels

% of SNP kept^1^	Duroc		Hampshire		Landrace		Yorkshire	
	
	average *r^2 ^*^2^	average distance in kb ^2^	average *r^2 ^*^2^	average distance in kb ^3^	average *r^2 ^*^2^	average distance in kb ^3^	average *r^2 ^*^2^	average distance in kb ^3^
50%	0.40	141	0.37	148	0.30	120	0.34	123
25%	0.34	281	0.31	296	0.25	239	0.28	246
10%	0.25	703	0.23	740	0.20	597	0.21	613
2%	0.10	3,507	0.11	3,693	0.09	2,978	0.09	3,056
1%	0.05	7,026	0.06	7,399	0.05	5,963	0.05	6,127
0.5%	0.02	14,120	0.04	14,872	0.03	11,977	0.02	12,313

^1^Percentage of SNP included in the current set of markers

^2^Average *r^2 ^*for SNP with adjacent map positions for the current set of markers

^3^Average distance in kb for SNP with adjacent map positions in the current set of markers

### Persistence of Phase

**Figure 2:**
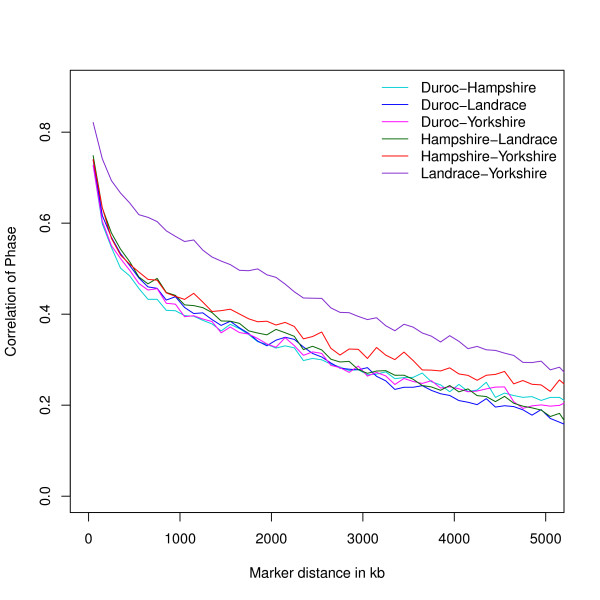
**Correlation of gametic phase compared across breeds over distance**. Correlation of Phase between breeds for SNP pairs grouped by distance in intervals 100 kb long covering 0 to 5 Mb across the genome.

Persistence of phase is a measure of the degree of agreement of LD phase for pairs of SNP between two populations. To estimate persistence of phase, we calculated *r_ij _*as the square root of *r_ij_^2 ^*in equation (1) between all possible combinations of SNP *i *and *j *respectively, using the sign of the non-squared numerator. If *r^2 ^*between two markers is equal in two populations, but their corresponding *r *has opposite sign, the gametic phase is reversed [16]. Persistence of phase over a certain genomic distance interval can be estimated as the pairwise Pearson correlation coefficient (*R_k, k'_*) of inter-marker *r_ij _*between two populations *k *and *k' *(Equation 3). For all pairwise comparisons of breeds we estimated *R_k, k' _*and the percentage of SNP with reversed sign of *r*. Similar to our computation of average *r^2^*, we grouped SNP pairs in classes of inter-marker distances 100 kb long and computed persistence of phase within each class starting at 0 up to 10 Mb (Figure 2). In theory, the Pearson correlation coefficient ranges between -1 and 1. Large negative values are a result of high LD (*r^2^*) in both breeds but phase is reversed between them. High positive values are a result of high *r^2 ^*and equal phase in both breeds [16]. Correlation of phase between SNP less than 100 kb apart ranged from 0.73 for Duroc with Hampshire and Yorkshire to 0.82 for Landrace with Yorkshire. Considering SNP pairs with an average distance of 0.9 to 1 Mb, correlation of phase decreased to 0.41 for Duroc with Hampshire and to 0.57 for Yorkshire with Landrace (Table 3). Persistence of phase decreased with increasing marker distance at a rate comparable to that observed for the decrease in average *r^2 ^*with increasing marker spacing. The slope of the decline was lower for the correlation between Landrace and Yorkshire when compared to other breed comparisons. Applying a z-test with Fishers' transformation [25] to the correlation of phase at < 10 kb, the correlation of phase between Landrace and Yorkshire was significantly larger (*p < 0.001, n = 1,520*) than all other breed combinations. Results for the correlation of phase were not significantly different (*p > 0.05, n = 1,520*) in the Duroc-Hampshire, Duroc-Landrace, Duroc-Yorkshire, Hampshire-Yorkshire, and Hampshire-Landrace pairings (Table 3). For these five population comparisons, the average proportion of SNP with *r *having opposite sign ranged between 9-11% for SNP spaced within 10 kb and up to 45-49% for SNP spaced between 4.9 and 5 Mb (Table 3). In general, the estimates of *r *with reversed sign for Landrace-Yorkshire were lower ranging from 9% to 45%. These results suggested a closer population relationship between the Landrace and Yorkshire populations than among all other populations.

**Table 3:** Pairwise breed comparison of correlation of phase and proportion of phase agreement at various distances

Breeds Compared	Distance^1^	Proportion of *r *with opposite sign^2^	Correlation of *r_ij(k) _*and *r_ij(k')_*^3^
	0-10 kb	0.107	0.875
	10-50 kb	0.184	0.762
Duroc - Hampshire	50-100 kb	0.246	0.668
	0.9-1 Mb	0.391	0.408
	4.9-5 Mb	0.469	0.210

	0-10 kb	0.108	0.872
	10-50 kb	0.186	0.773
Duroc - Landrace	50-100 kb	0.251	0.681
	0.9-1 Mb	0.395	0.438
	4.9-5 Mb	0.485	0.190

	0-10 kb	0.104	0.870
	10-50 kb	0.195	0.761
Duroc - Yorkshire	50-100 kb	0.252	0.670
	0.9-1 Mb	0.396	0.422
	4.9-5 Mb	0.468	0.201

	0-10 kb	0.099	0.882
	10-50 kb	0.184	0.776
Hampshire - Landrace	50-100 kb	0.242	0.697
	0.9-1 Mb	0.392	0.441
	4.9-5 Mb	0.475	0.189

	0-10 kb	0.113	0.871
	10-50 kb	0.189	0.771
Hampshire - Yorkshire	50-100 kb	0.249	0.686
	0.9-1 Mb	0.389	0.439
	4.9-5 Mb	0.459	0.245

	0-10 kb	0.087	0.921
	10-50 kb	0.160	0.842
Landrace - Yorkshire	50-100 kb	0.204	0.783
	0.9-1 Mb	0.353	0.571
	4.9-5 Mb	0.448	0.297

^1^Interval length in kb

^2^Proportion of SNP pairs having *r *with reversed sign within the interval

^3^Correlation of phase between two breeds (*k *and *k'*) within the given interval

## Discussion

### Extent of Linkage Disequilibrium

Current effective population size of the breeds used in this study was previously estimated, using pedigree information, to be between 74 (Landrace) and 113 (Duroc, Yorkshire) [26]. Consistent with having the largest current effective population size, we find that long range *r^2 ^*(10 Mb, Figure 1) estimated from our data was smallest for Duroc and Yorkshire (0.035, 0.03). In Hampshire, a smaller effective population of 109 corresponded to higher *r^2 ^*at 10 Mb (0.046). Due to the similar long range *r^2 ^*(0.035) at 10 Mb we would have expected the Landrace population to have an effective population size comparable to that of Duroc and Yorkshire. However, using pedigree data Welsh et al. [26] estimated the current effective population size of Landrace to be 74. The reason for this discrepancy remains unknown. Several previous studies investigated LD in pigs using reduced numbers of microsatellite markers and fewer animals from commercial populations [17,27]. Nsengimana et al. [27] found relatively large estimates of LD (*D'*) from 0.29 to 0.41 using 15 microsatellite markers. In contrast, using *r^2 ^*instead of *D' *and thereby correcting for minor allele frequency, Harmegnies et al. [17] found *r^2 ^*ranging from 0.15 to 0.19 for marker distance < 1 cM and 0.10-0.12 for markers spaced between 1 cM and 5 cM, using 29 microsatellite markers on SSC15, comparable to our results of *r^2 ^*between 0.16-0.22 for markers spaced between 1 and 5 Mb. Du et al. [28] estimated *r^2 ^*from 4,500 SNP markers in six commercial lines of pigs and found estimates of average *r^2 ^*= 0.51 for markers less than 0.1 cM apart, and estimates of 0.21 and 0.07 at marker distances of 1 cM and 5 cM respectively. Similarly, our populations had average *r^2 ^*of 0.15 to 0.20 and 0.05 to 0.08 at marker distances of 1 Mb and 5 Mb, respectively. A recent study conducted by Uimari & Tapio [16] used the same genotyping platform as our study to estimate *r^2 ^*and effective population size in Finnish Landrace and Yorkshire populations. Uimari & Tapio [16] found average *r^2 ^*of 0.43 and 0.46 for adjacent markers in the Finnish Landrace and Yorkshire populations, respectively, which was higher than our results of 0.36 for Landrace and 0.39 for Yorkshire. In addition, Uimari & Tapio reported that the *r^2 ^*for markers spaced at 5 Mb decreased to 0.09 and 0.12 in the Finnish Landrace and Yorkshire breeds, respectively. In the present study, *r^2 ^*declined further to 0.05-0.06 at 5 Mb marker spacing for Landrace and Yorkshire (Table 1). The higher average *r^2 ^*for distant (*r^2 ^*> 0.2 for 1 Mb) markers in the Finnish populations could be explained by smaller effective population size of the Finnish populations, causing higher *r^2 ^*on average. This is partially confirmed by comparing the estimated effective size of the Finnish populations (*N_e _*= 91, 61 for Landrace and Yorkshire, respectively) [16], to estimated effective population sizes of the populations used in the current study reported by Welsh et al. [26] (*N_e _*= 74, 113 for Landrace and Yorkshire, respectively), where the current effective population size for Finnish Yorkshire is approximately half that of our Yorkshire population. Compared to recent estimates from Canadian populations we found estimates of average *r^2 ^*for markers with pairwise distance below 100 kb to be consistent in Landrace (US: 0.34, Canadian: 0.31) and Yorkshire (US: 0.37, Canadian: 0.32) [29]. However, in Duroc estimates of average *r^2 ^*for markers with pairwise distance below 100 kb were considerably higher in the US population (0.42) compared to the Canadian population (0.31) [29].

### Persistence of Phase

Persistence of phase can be used to infer upon the history of a species and relatedness of breeds within that species as well as on reliability of across population GWA and GEVB prediction [14]. Persistence of phase was previously reported for three Canadian swine breeds (Duroc, Landrace, Yorkshire) [29]. For SNP with pairwise distance below 50 kb we estimated persistence of phase to be 0.88 between Landrace and Yorkshire and 0.82 for both Landrace and Yorkshire with Duroc. In the Canadian breeds persistence of phase also indicates a closer relationship between Landrace and Yorkshire (0.82) and a more distant relationship between Landrace/Yorkshire and Duroc [29]. We found correlation of phase of 0.82 for Landrace/Yorkshire with Duroc, while the Canadian breeds had 0.66/0.65, indicating less agreement of phase even at short pairwise distance [29]. Our results showed that correlation of phase for the pig breeds in this study ranged between 0.87 for Duroc-Yorkshire and 0.92 for Landrace-Yorkshire for markers with pairwise distance < 10 kb. Previous research in Australian cattle breeds [14] showed correlation of phase between 0.68 for Australian Angus-New Zealand Jersey to 0.97 for Dutch Holstein-Black and White. At increasing marker distance, correlation of phase for the pig breeds in this study decreased (range in *r*: 0.41 to 0.57 at an average pairwise marker distance of 1 Mb). This decrease however was less than the decrease de Roos et al. [14] observed in all but two of the cattle breeds they considered (< 0.4 for markers spaced 1 Mb). While correlation of phase was similar between these pig breeds and dairy cattle at short range (< 10 kb), the pig breeds showed generally larger correlation of phase than the dairy cattle [14] at increasing marker distances.

If two populations diverged from a common ancestral population, their correlation of phase can be expressed as *r_0_^2^(1-c)^2T^*, where *r_0_^2 ^*is a measure of LD in the common ancestral population, *c *is the recombination distance between markers and *T *is time since breed divergence in generations [30]. For markers as close as 10 kb the recombination distance *c *will be almost 0, so that correlation of phase at those short distances can serve as an estimation of *r_0_^2 ^*in the common ancestral population. Since correlation of phase was comparable in the pig populations (0.87-0.92) for markers with pairwise distance below 10 kb to that reported in Australian cattle (0.80-0.97) [14], LD in the common ancestral pig population is likely to be similar to that in the common ancestral population of Australian cattle breeds. Larger correlation of phase at increasing marker distance (1 Mb) in the pig populations used in this study (0.41-0.57) compared to Australian cattle breeds (< 0.40) suggests that *T *is smaller in our pig breeds than it is in the cattle breeds. The expected correlation of *r *between two breeds can be expressed as *e^-2cT ^*[14]. To estimate the time since breed divergence for the pig breeds in this study we used SNP with pairwise distance between 10 kb and 300 kb, and estimated correlation of phase for each 2.5 kb interval. We calculated the linear regression of the natural logarithm of the estimated correlation of phase onto the average pairwise distance *c*. The slope of this regression is an estimate of *-2T*. Consequently, the slope divided by *-2 *is the number of generations (*T*) since these two breeds have diverged [14]. Results suggest that the pig breeds in this study diverged approximately 40-66 generations ago. The expected correlation of phase would decrease to 0.41 and 0.02 at 1 cM and 5 cM distance respectively in the Yorkshire-Landrace comparison, assuming *T *of 40 and *r_0_^2 ^*of 0.92. We observed a correlation of phase of 0.57 and 0.30 at 1 Mb and 5 Mb, respectively, between these two breeds, indicating that a *T *of 40 may overestimate the actual time since breed divergence. One possible cause of this observation is admixture between these two breeds, causing more common LD between them than what would be expected from fully diverged breeds [14]. We obtained the date of herd book closure for each of the breeds in this study, and assuming a generation interval of approximately 2 years [26], Duroc, Hampshire, Landrace, and Yorkshire have existed as distinct breeds for at least 38.5, 44.5, 31.5, and 30.5 generations, respectively. The time of herd book closure does not directly indicate the time since breed divergence, since distinguishable breeds must have existed before herd book closure. Nevertheless, the time of herd book closure further supports our observation that Landrace and Yorkshire have developed as separate breeds later than Duroc and Hampshire.

### Implications of estimated levels of LD for GEBV implementation

Our results have several important implications for future implementation of genomic selection in swine. Accuracy of prediction of genome wide marker assisted selection can be directly affected by the chosen marker density (resulting in average *r^2 ^*between markers and QTL), and the size of the training population [8].

The currently used marker panel, containing approximately 40,000 usable markers, had average *r^2 ^*of approximately 0.4 between adjacent markers for all four breeds. That exceeds the level of *r^2 ^*= 0.2 simulated by Meuwissen et al. [19] to reach prediction GEVB accuracy around 0.85. Furthermore, our results indicated that reducing the original marker panel to 10% of the markers (3,000-4,000 SNP) still resulted in average *r^2 ^*for adjacent markers exceeding 0.2 in all four breeds. On the other hand, recent research in Australian Holstein Friesian cattle has shown [31] that using subsets of 3000-5000 SNP to estimate direct genomic breeding values (DGV) could only reach 80% of the prediction accuracy previously estimated using approximately 42,000 SNP. Such a reduction in prediction accuracy will be unacceptable for most practical implementations. However, the accuracy of GEBV predicted by low density panels can be increased through the use of genotype imputation [32], where high density genotypes are imputed using low density SNP genotypes and a high density reference panel of haplotypes [24]. Weigel et al. [33] used approximately 10% of 2,693 SNP from Bos Taurus chromosome 1 to impute the full SNP set in a Jersey population. They found that using a high density reference genotype panel (n = 2,542 animals), the imputation accuracy of the non-typed markers was between 0.86 and 0.94. Average *r^2 ^*in our populations ranged from 0.36 to 0.48 for markers less than 100 kb apart, comparable to average *r^2 ^*= 0.38 for markers spaced at < 100 kb in the Jersey population [34]. Assuming a comparable decline of LD for increasing marker distance between the Jersey population and our pig populations, we would expect to accurately impute approximately 90% of the high density genotypes, using a low density panel containing 10% of the markers. More recent results reported even higher average accuracy of imputation (approximately 95%) when imputing 42,000 SNP in the Bovine 50 K using the 3 K subset in Holstein cattle [35]. To assess the accuracy of GEBV estimated from imputed genotypes Weigel et al. [32], used the same Jersey population from their previous study [33], and they found that the accuracy of GEBV based on imputed markers was 95% of the accuracy of the GEBV estimated using the observed genotypes [32]. As noted above average *r^2 ^*is similar between the American Jersey population and our pig populations, suggesting that future research in genomic selection in swine should explore the use of imputed low density genotypes to increase cost efficiency. Previous research in humans [36], and European Holstein cattle [37] indicated that combining haplotypes from closely related populations can increase the accuracy of genotype imputation, while research in sheep suggests that breed specific reference haplotypes would yield better accuracy [38]. The success of combined haplotypes for genotype imputation depends on the relatedness between the populations. Further research is necessary to determine if persistence of phase is large enough in our pig populations to increase imputation accuracy when combining reference haplotypes across breeds. As noted by Goddard [18], the accuracy of GEBV prediction can be expressed as a function of the LD between marker and QTL and the accuracy of estimated SNP effects. The loss in accuracy of GEVB prediction caused by imputing instead of observing genotypes could be compensated by increasing the number of animals used to estimate SNP effects. If not enough animals are available for the estimation of SNP effects, animals from different, but closely related, populations could be combined to estimate SNP effects for GEBV prediction in both populations [13,39]. The squared short-range (< 10 kb) correlation of phase can also serve as the accuracy with which we can predict a marker-QTL association in one population using known marker-QTL associations from another population. For the pig breeds reported in this study the squared correlation of phase for close markers (0-100 kb) ranged from 0.53 to 0.67. To evaluate whether these accuracies would warrant the use of a combined training population to estimate SNP effects accurately for both populations we refer to a simulation study conducted by de Roos et al. [40] estimating the accuracy of GEBV prediction for combined training populations of highly, moderately and lowly related populations. Correlation of phase for populations diverged approximately *T=*30 generations ago was reported to be below 0.80 for markers with pairwise distance below 0.055 cM [40]. We found correlation of phase between Landrace-Yorkshire of around 0.80 at a corresponding marker distance. De Roos et al. [40] concluded that reliability of GEBV prediction could be increased between 0.05-0.10 points in two populations, when approximately 40,000 marker genotypes are available, heritability is *h^2^=*0.3 or higher, 1000 animals from each population were used to estimate SNP effects, and under the assumption that QTL effects are the same for both populations [40]. In addition, they found that for genetically distant populations, at least 1,000 animals with genotypes and phenotypes available in each population were needed to avoid a decrease in the reliability of prediction [40]. When SNP effects estimated in one population are used to calculate GEBV for another population which diverged approximately *T=*30 generations ago, the reliability of the predicted GEBV was 0.65 assuming both high marker density (M = 40,000) and heritability *h^2 ^*= 1 [40]. Consequently, combining animals into a multi-breed panel to estimate SNP effects is likely to be only marginally beneficial for the pig breeds in this study, given the estimated correlation of phase and the large number of animals and markers required [40].

## Conclusions

We used the PorcineSNP60 chip [12] to obtain high density genotypes (34,000-40,000 SNP) from pig trios in four breeds. From this data we estimated *r^2 ^*as a measure of LD across the genome as well as correlation of *r*, which measures phase agreement between breeds. We found *r^2 ^*of approximately 0.4 for markers less than 100 kb apart, which is higher than comparable estimates reported for North American Holstein cattle [15] as well as various Australian cattle breeds [14]. The same was true for average *r^2 ^*between markers with pairwise distance larger than 1 Mb, indicating a smaller past effective population size of these pig breeds. We also report a relatively slow rate of decay of LD over distance, observing *r^2 ^*around 0.2 at 1 Mb. The comparably high long range LD is an indicator that good accuracy can be expected for future implementations of GEBV in pigs using 10% (3,000-4,000) of the SNP used in the current assay or less, along with genotype imputation. We would encourage future research in genomic selection in swine to especially focus on the possible benefits of the combined use of reduced marker panels and genotype imputation. To successfully promote the use of genomic selection in swine it will be necessary to increase cost efficiency while maintaining high accuracy of prediction. Currently no low density panels for SNP genotyping are publicly available for swine, but the presented results will be available to aid in the development of efficient SNP platforms. Relatively low persistence of phase reported here implies that the use of multi-breed panels estimating SNP effects for genomic selection will likely be limited, especially when using low density genotypes, but the merit of combining reference haplotypes for genotype imputation should be further investigated.

## Methods

### Sample Design

For this study sire/dam/offspring trios of the Duroc, Hampshire, Landrace and Yorkshire breeds were selected from the National Swine Registry (NSR) pedigree. Selected parents were unrelated for at least two generations. All animals were genotyped using the Illumina PorcineSNP60 (Number of markers *M *= 62,163) Genotyping BeadChip (Illumina Inc.) [12] at a commercial laboratory (GeneSeek, a Neogen Company, Lincoln, NE). All SNP showing Mendelian inconsistencies for a trio were set missing in that particular trio. If one or more animals within a trio had missing genotypes in more than 10% of the SNP that trio was eliminated from further analysis. Similarly, SNP were removed if they did not have genotypes available for at least 90% of the samples across all breeds (*M_CallRate < 0.9 _*= 5080). Only autosomal SNP were considered in this study, leading to the exclusion of all SNP with an uncertain map position on build 10 of the pig genome sequence, as well as SNP on the sex chromosomes (*M_non-autosomal _*= 9308). To exclude non-segregating SNP from the analysis we removed markers with minor allele frequency (MAF) below 5% within each breed separately. The number of fixed SNP varied substantially between breeds: we excluded *M_MAF < 5% _*= 13,646 SNP in Duroc, *M_MAF < 5% _*= 15,405 SNP in Hampshire, *M_MAF < 5% _*= 7,631 SNP in Landrace and, *M_MAF < 5% _*= 8,665 SNP in Yorkshire. Additionally, SNP were excluded for failure to meet Hardy Weinberg Equilibrium (*p < 0.001*) within breeds causing *M_HWE < 0.001 _*= 117, 85, 146, and 176 SNP to be discarded in Duroc, Hampshire, Landrace, and Yorkshire respectively. After applying the described filtering criteria, a total of 30, 26, 29, and 32 trios were included for the Duroc, Hampshire, Landrace and Yorkshire breeds, respectively. And a total of 34,129, 32,370, 40,144 and 39,110 SNP spaced at an average distance of 70, 74, 60 and 61 kb satisfied the SNP selection criteria for Duroc, Hampshire, Landrace and Yorkshire, respectively.

### Estimation of average LD and persistence of phase

Haplotypes were obtained for the founder animals using the trio option of BEAGLE [24], phasing the genotypes by chromosome. Sampling animals in trios was shown to yield improved accuracy of estimated haplotypes [41]. To further increase haplotype accuracy, BEAGLE was set to run 100 iterations of the phasing algorithm and sample 100 haplotype pairs for each individual per iteration. Additionally, a short simulation experiment was conducted showing that for MAF above 5% average *r^2 ^*can be reliably estimated from the current sample size (results not shown). Alleles for each SNP were re-coded as 0/1, keeping the reference allele constant across all four populations, allowing for later determination of phase agreement. Haplotypes and code needed to reproduce these results are publicly available at https://www.msu.edu/~steibelj/JP_files/LD_estimate.html.

For all pairs of SNP *r^2 ^*was estimated, using allelic frequencies of the founding animals, according to the following equation:

(1) $$r_{ij}^{2}=\frac{{\left(p_{ij}-p_{i}\cdot p_{j}\right)}^{2}}{p_{i}\cdot \left(1-p_{i}\right)\cdot p_{j}\cdot \left(1-p_{j}\right)}$$

where *p_i_, p_j _*are the marginal allelic frequencies at the *i^th ^*and *j^th ^*SNP respectively and *p_ij _*is the frequency of the two-marker haplotype [42], using the freely available software R [43]. Marker pairs were grouped by their pairwise physical distance into intervals of 100 kb starting from 0 up to 10 Mb. Average *r^2 ^*for SNP pairs in each interval was estimated as the arithmetic mean of all $r_{ij}^{2}$ (Equation 1), with the pairwise distance between the *i^th ^*and *j^th ^*element of the currently considered interval:

(2) $${\bar{r}}^{2}=\frac{1}{\overset{18}{\underset{l=1}{\sum }}\left(M_{l}-1\right)}\overset{M_{l}-1}{\underset{i=1}{\sum }}r_{i,i+1}^{2},$$

where ${\bar{r}}^{2}$ is the average of all adjacent SNP across *18 *autosomes (*l*), with *M_l _*SNP per chromosome. To estimate average *r^2 ^*between adjacent markers for different marker densities a certain percentage of markers (50%, 75%, 90%, 95%, 99%, and 99.5%) were removed before average *r^2 ^*was estimated using equation (2). To select markers, an increasing proportion of SNP were sequentially removed solely considering their map position, so that for instance: to reduce a panel to 50%, every second marker was kept for analysis, for 25% every fourth was kept and so on.

To estimate persistence of phase only markers with minimum MAF of 5% in all breeds were included in the analysis, resulting in 22,340 common SNP across all breeds. Correlation of phase was estimated for intervals of 100 kb (from 0 to 10 Mb). We excluded markers with pairwise distance above10 Mb to decrease the computational load. Estimates of average *r^2 ^*at larger distances are close to zero, which would cause correlation of phase to be close to zero as well. Persistence of phase was then estimated as:

(3) $$R_{k,k^{\prime }}=\frac{\underset{\left(i,j\right)\in p}{\sum }\left(r_{ij\left(k\right)}-{\bar{r}}_{\left(k\right)}\right)\left(r_{ij\left(k^{\prime }\right)}-{\bar{r}}_{\left(k^{\prime }\right)}\right)}{S_{\left(k\right)}S_{\left(k^{\prime }\right)}}$$

where *R_k, k' _*is the correlation of phase between *r_ij(k) _*in population *k *and *r_ij(k') _*in population *k', s_(k) _*and *s_(k') _*are the standard deviation of *r_ij(k) _*and *r_ij(k') _*respectively, and ${\bar{r}}_{\left(k\right)}/{\bar{r}}_{\left(k^{\prime }\right)}$ are the average *r_ij _*across all SNP *i *and *j *within interval *p *for population *k *and *k' *respectively.

## Authors' contributions

JPS, ROB, CS, and CWE designed the experiments. CS and ROB identified trios for data collection and oversaw the collection of tissue samples. CWE coordinated the genotyping. JPS and YMB completed statistical analysis and wrote the manuscript. All authors read and approved the paper.
